# The Relationship between Depressive Symptoms, Quality of Life and miRNAs 8 Years after Bariatric Surgery

**DOI:** 10.3390/nu15194109

**Published:** 2023-09-22

**Authors:** Virginia Mela, Zaida Agüera, Maria D. Alvarez-Bermudez, Flores Martín-Reyes, Roser Granero, Ana Sánchez-García, Wilfredo Oliva-Olivera, Monica Tomé, Francisco J. Moreno-Ruiz, Rocío Soler-Humanes, Jose L. Fernández-Serrano, Pilar Sánchez-Gallegos, Jose M. Martínez-Moreno, Raquel Sancho-Marín, Fernando Fernández-Aranda, Eduardo García-Fuentes, Francisco J. Tinahones, Lourdes Garrido-Sánchez

**Affiliations:** 1 Instituto de Investigación Biomédica de Málaga y Plataforma en Nanomedicina-IBIMA Plataforma BIONAND, 29590 Malaga, Spain; virginia.mela@ibima.eu (V.M.); mar17alv@gmail.com (M.D.A.-B.); floresmarey@hotmail.com (F.M.-R.); anasanchez-alozaina@hotmail.com (A.S.-G.); oliva_olivera@hotmail.com (W.O.-O.); lourgarrido@gmail.com (L.G.-S.); 2 Department of Medicine and Dermatology, Faculty of Medicine, University of Malaga, 29010 Malaga, Spain; 3 Unidad de Gestión Clínica de Endocrinología y Nutrición, Hospital Universitario Virgen de la Victoria, 29010 Malaga, Spain; 4 CIBER Fisiopatología de la Obesidad y Nutrición (CIBERObn), Instituto Salud Carlos III, 28029 Madrid, Spain; zaguera@ub.edu (Z.A.); roser.granero@uab.cat (R.G.); ffernandez@bellvitgehospital.cat (F.F.-A.); 5 Departament d’Infermeria de Salut Pública, Salut Mental i Maternoinfantil, Escola d’Infermeria, Facultat de Medicina i Ciències de la Salut, Universitat de Barcelona, 08036 Barcelona, Spain; 6 Psychoneurobiology of Eating and Addictive Behaviors Group, Neurosciences Programme, Bellvitge Biomedical Research Institute (IDIBELL), 08908 Barcelona, Spain; 7 Unidad de Gestión Clínica de Aparato Digestivo, Hospital Universitario Virgen de la Victoria, 29010 Malaga, Spain; 8 Department of Psychobiology and Methodology, Autonomous University of Barcelona, 08193 Barcelona, Spain; 9 Unidad de Gestión Clínica de Endocrinología y Nutrición, Hospital Regional Universitario de Málaga, 29009 Malaga, Spain; montome@hotmail.com; 10 Unidad de Gestión Clínica de Cirugía General y Digestiva, Hospital Regional Universitario de Málaga, 29010 Malaga, Spain; javier.morenoruiz@gmail.com; 11 Unidad de Gestión Clínica de Cirugía General y Digestiva, Hospital Universitario Virgen de la Victoria, 29010 Malaga, Spain; rocioshumanes@hotmail.com (R.S.-H.); jlfserrano@telefonica.net (J.L.F.-S.); 12 Department of Surgical Specialities, Biochemistry and Immunology, Faculty of Medicine, University of Malaga, 29010 Malaga, Spain; pilars@uma.es (P.S.-G.); jmmartinezm@uma.es (J.M.M.-M.); raquelsanchomarin@gmail.com (R.S.-M.); 13 Department of Psychiatry, University Hospital of Bellvitge, 08907 Barcelona, Spain; 14 Department of Clinical Sciences, School of Medicine and Health Sciences, University of Barcelona, 08907 Barcelona, Spain; 15 CIBER Enfermedades Hepáticas y Digestivas (CIBERehd), Instituto Salud Carlos III, 28029 Madrid, Spain

**Keywords:** bariatric surgery, class III obesity, quality of life, depressive symptoms, miRNAs

## Abstract

(1) Background: There are conflicting results on whether weight loss after bariatric surgery (BS) might be associated with quality of life (QoL)/depressive symptomatology. We aim to determine whether BS outcomes are associated with QoL/depressive symptomatology in studied patients at the 8-year follow-up after BS, as well as their relationship with different serum proteins and miRNAs. (2) Methods: A total of 53 patients with class III obesity who underwent BS, and then classified into “good responders” and “non-responders” depending on the percentage of excess weight lost (%EWL) 8 years after BS (%EWL ≥ 50% and %EWL < 50%, respectively), were included. Basal serum miRNAs and different proteins were analysed, and patients completed tests to evaluate QoL/depressive symptomatology at 8 years after BS. (3) Results: The good responders group showed higher scores on SF-36 scales of physical functioning, role functioning—physical, role functioning—emotional, body pain and global general health compared with the non-responders. The expression of hsa-miR-101-3p, hsa-miR-15a-5p, hsa-miR-29c-3p, hsa-miR-144-3p and hsa-miR-19b-3p were lower in non-responders. Hsa-miR-19b-3p was the variable associated with the response to BS in a logistic regression model. (4) Conclusions: The mental health of patients after BS is limited by the success of the intervention. In addition, the expression of basal serum miRNAs related to depression/anxiety could predict the success of BS.

## 1. Introduction

The prevalence of obesity has tripled worldwide in recent decades [1]. Among all treatment options, bariatric surgery (BS) is the most effective, not only in reducing body weight but also in decreasing the risk of developing obesity-related comorbidities [2,3]. Roux-en-Y gastric bypass (RYGB) and biliopancreatic diversion (BPD) promote weight loss through malabsorption of ingested calories and are more effective in terms of losing weight compared with sleeve gastrectomy (SG) [3,4]. BS is considered a successful intervention when the outcome of the surgery is the loss of ≥50% of the percentage of excess weight (%EWL), which is related to decreasing the risk of developing obesity-related diseases [5]. This index is widely used, even in recent studies [6,7,8,9,10], including reviews on this topic [11,12,13], to analyse the efficacy of bariatric surgery on weight loss. However, there is still a high percentage of failure (up to 50%), since the efficacy of the procedure depends on many factors, such as type of procedure, age, sex and ethnic background [14]. For example, a recent study showed that SG was associated with a higher risk of weight non-response, measured by the %EWL, compared with gastric bypass in a total of 60,426 individuals [6]. This lack of response to different types of bariatric procedures (such as SG), primary vertical banded gastroplasty and gastric band procedures, leads in many cases to the consideration of a reconversion. Revisional RYGB is commonly employed to revise bariatric procedures due to insufficient WL or weight recurrence. However, despite this, only one third of all the patients undergoing revisional surgery had a sufficient %EWL after 2 years compared with primary RYGB [11].

Obesity is often accompanied by psychological distress and decreased quality of life (QoL) [15]. Depression and anxiety are commonly comorbid conditions in patients with obesity, mainly in those enrolled in BS procedures [16]. Most of the literature shows that BS has been shown to improve the QoL [17,18]. It is well known that psychosocial and psychiatric factors are predictors of BS outcome [19]. However, it is so far unclear which factors best predict a successful BS and in what way they act. While some studies found that presurgical depression may act as a predictor of greater weight loss after BS as measured by the %EWL [20], others, in contrast, revealed a negative association between depression and a favourable outcome at the 2-year follow-up [21]. These conflicting results highlight the need to explore this issue further. Most of the studies on this topic show a positive effect of BS on the psychological state of these patients, since after BS, they recover physical, mental and social functions [17]. In this context, the BS outcome could be associated with QoL or depressive symptomatology. However, there is a limited amount of research on long-term outcomes in terms of weight loss, resolution of co-morbidities and QoL after BS. In a previous study, RYGB was shown to lead to positive results regarding the %EWL and improved QoL at more than 10 years follow-up [10]. However, post-intervention improvements in QoL diminished over time and were dependent on the %EWL [7].

Because obesity or its perception is psychologically stressful [22], obesity is also a chronic psychological stressor exaggerating stress-dependent psychoimmune–neuroendocrine network dysfunction [23]. Depression could be linked to obesity through different physiological pathways including metabolic and mood regulation via adipokines, the autonomic nervous system and different hormones, which are linked by the hypothalamic–pituitary–adrenal axis and immuno-inflammatory function [24]. One interesting fact is the relation between different cytokines and the depression state. Some cytokines, such as IL-1β, IL-6 and tumour necrosis factor-α (TNF-α) appear to be involved in the onset and progression of depression [25]. Cytokines, such as interferon-γ (IFNγ), *C*-reactive protein (CRP) and IL-6, increase with chronic stress [26], correlate with the severity of depression [27] and predict cognitive changes in depression [28]. Cytokines can enter the brain through the blood–brain barrier and affect the neuron function and neuroactive molecule production, which are associated with depression [29]. Neuroinflammation could be the link between obesity and depression, as obesity leads to a low-grade inflammation state, at least in animals [30]. Increased inflammatory cytokines in the brain and in serum, such as CRP, and soluble cell-adhesion molecules IL-6, IL-1β, TNF-α and NF-κB, would be involved in the susceptibility to depression in stressed obese mice [30]. However, other serum proteins related to adipose tissue have not been well studied and may be involved in the relationship between obesity and depression. Several studies suggest the involvement of white adipose tissue in the depression–neuroinflammatory pathways [31], which are a mix of adipocytes, immune, vascular stromal and nervous cells.

Other systemic molecules that could be linked to obesity, including mental health aspects, are the so-called “miRNAs”. miRNAs can regulate gene expression by post-transcriptional repression of their mRNA targets. miRNAs have been studied in relation to different pathologies, including obesity and depression [32,33]. Recent systematic reviews identified different circulatory miRNAs that were dysregulated in depression, most of them upregulated, such as miR-451a and miR-124-3p [32,34]. Moreover, other upregulated miRNAs, such as miR-34a-5p, appear to be susceptible to the action of 7-chlorokynurenic acid (7-CTKA), a potential and rapid antidepressant [32]. Other systematic reviews on circulatory obesity-related miRNAs found that most of them were also upregulated, such as miR-26b-5p and miR-30d-5p [33]. Some of these miRNAs could be associated with the mental health of patients 8 years after BS. However, to our knowledge, there are no studies analysing the profile of circulating miRNAs associated with depression in obesity 8 years after BS.

Therefore, in the present study, we aimed at achieving the following objectives: (a) to determine whether BS outcome is associated with QoL or depressive symptomatology in the studied patients at the 8-year follow-up after BS and (b) to analyse the relationship between different serum proteins and miRNAs and mental health and BS outcome.

## 2. Material and Methods

### 2.1. Participants

Ninety patients with class III obesity (BMI > 40 kg/m^2^), who were scheduled for BS at the “Virgen de la Victoria University Hospital” and “Regional University Hospital” in Malaga between 2010 to 2013, were invited to participate in the present retrospective study. Participants were divided into 2 groups based on the surgical procedure: RYGB and BPD (both restrictive and malabsorptive techniques) (*n* = 41) vs. SG (restrictive technique) (*n* = 49). Among these 90 patients, only those who completed psychometric tests at the 8-year follow-up after the intervention were included in this study (*n* = 53), namely, 36 patients who underwent RYGB or BPD and 17 who underwent SG. In addition, these patients (*n* = 53) were classified according to their BS outcome into two groups: “good responders” (GR) (*n* = 30) vs. “non-responders” (NR) (*n* = 23). This classification was assessed by measuring the percentage of excess weight lost (%EWL) 8 years after BS. We assessed %EWL as 100 × (preoperative weight—weight at the time of evaluation)/(preoperative weight—weight corresponding to BMI = 25 kg/m^2^) [35]. The different patterns of weight loss were defined based on the EWL Reinhold criteria modified by Christou et al. [36]. The patients with %EWL > 50% at the follow-up were considered GR. On the other hand, patients with %EWL < 50% at the follow-up were considered NR. The percentage of GR and NR did not significantly vary between groups of included and excluded patients.

The following sociodemographic data were obtained from patients: sex, age, medical history and drug consumption. All patients underwent standardized anthropometric and biochemical examinations. The percentage of total weight loss (%TWL) was calculated as (Initial Weight–Post-Operative Weight) × 100/Initial Weight [37]. Exclusion criteria included patients under 18 or over 60 years old, tobacco or alcohol abuse, psychiatric diseases, cardiovascular disease, acute inflammatory disease and antibiotic or probiotic treatment. The study was carried out in accordance with the Code of Ethics of the World Medical Association (Declaration of Helsinki). All participants gave their written informed consent, and the study was reviewed and approved by the Malaga Provincial Research Ethics Committee (Malaga, Spain).

### 2.2. Psychometric Instruments

All participants completed the following questionnaires only at 8 years after BS:

- The 36-item Short Form Health Survey (SF-36) [38]. The SF-36 is a valid and self-administered questionnaire that assesses eight dimensions of QoL: (1) Physical functioning (PF; 10 items), (2) Role functioning—Physical (RP; 4 items), (3) Body pain (BP; 2 items), (4) General health perception (GH; 5 items), (5) Vitality (VT; 4 items), (6) Social functioning (SF; 2 items), (7) Role functioning—Emotional (RE; 3 items) and (8) Mental health (MH; 5 items). Each domain is scored from 0 to 100, with greater scores indicating better perceived QoL (physical or mental health conditions). Excellent internal consistency was found in the study sample (Cronbach’s α = 0.96 for the total scale).
- Beck Depression Inventory (BD-II) [39]. This is a 21-item self-reported questionnaire that assesses the severity of depression symptoms. The total score can range from 0 to 63, with higher scores indicative of more severe depression symptoms. The clinical cut-off scores were classified as follows: no depression (0–13), mild depression (14–19), moderate depression (20–28) and severe depression (29 or more). In the current sample, total scores showed an excellent internal consistency (Cronbach’s α = 0.96).

### 2.3. Biochemical Data

Blood samples at baseline and 8 years after BS were collected after 12 h fasting. Serum was aliquoted and immediately stored at −80 °C. Serum glucose, cholesterol, triglycerides and HDL were analysed using an Advia Chemistry XPT autoanalyzer (Siemens Healthcare Diagnostics, Malvern, PA, USA). The LDL was calculated using the Friedewald equation. Serum insulin levels were measured via immunoassay using an ADVIA Centaur autoanalyzer (Siemens Healthcare Diagnostics, Malvern, PA, USA). Serum proteins, such as adhesion molecules (L-selectin, PECAM, ICAM-1, VCAM-1), metalloproteinases (MMP3, MMP9), growth factors (VEGF-A VEGF-D), adipokines (adiponectin, leptin), macrophage-related proteins (CD14, CD44, MBL, LVYE1) and other proteins (lactoferrin, elafin, NGAL, NRP1, PAI-1, RANTES) were analysed using ProcartaPlex immunoassay kit (Thermo Fisher Scientific Inc., Rockford, IL, USA) at baseline and 8 years after BS.

### 2.4. miRNAs Sequencing

Total RNA with an enhanced miRNA enrichment was extracted from baseline serum samples using the Maxwell^®^ 16 miRNA Tissue Kit (Promega, Madison, WI, USA). Total RNA concentration was measured using the Qubit^®^ RNA Assay Kit in the Qubit^®^ 3.0 Flurometer (Thermo Fisher Scientific Inc., Waltham, MA, USA). RNA integrity was assessed using the RNA Nano 6000 Assay Kit with the Agilent Bioanalyzer 2100 system (Agilent Technologies, Santa Clara, CA, USA). For next-generation sequencing (NGS), small RNA transcripts were converted into barcoded cDNA libraries using 400–500 ng of RNA per sample as input material for the small RNA library. Sequencing libraries were generated using NEBNext Multiplex Small RNA Library Prep Set for Illumina (NEB, MA) following the manufacturer’s instructions. Individual libraries, prepared with unique indexes, were pooled and subjected to the Illumina sequencing pipeline, passing through clonal cluster generation on sequencing-by-synthesis 1× 50–75 pb single-end reads on the NextSeq 550 (Illumina Inc., San Diego, CA, USA). From the total miRNAs obtained in the massive sequencing, we selected those miRNAs that related with depression and anxiety for this study according to previous studies [32,40,41]. To do so, we listed all miRNAs that were commented on in three studies referenced in our bibliography [32,40,41]. On the other hand, we had another list with miRNAs obtained through massive sequencing with detectable expression. These two miRNA lists were overlapped, obtaining the shared miRNAs. These shared miRNAs are the 19 miRNAs included in our study: hsa-miR-101-3p, hsa-miR-144-3p, hsa -miR-144-5p, hsa-miR15a-5p, hsa-miR-197-3p, hsa-miR-19a-3p, hsa-miR-19b-3p, hsa-miR-21-5p, hsa-miR-221-3p, hsa-miR-26b-5p, hsa-miR-29c-3p, hsa-miR-30a-5p, hsa-miR-30d-5p, hsa-miR335-5p, hsa-miR-484, hsa-miR-7704, hsa-miR-92b-3p, hsa-miR-338-3p and hsa-miR-338-5p.

### 2.5. Statistical Analysis

Results are expressed as mean values ± standard deviation (SD). After testing the normal distribution of continuous variables via the Shapiro–Wilk test, we applied logarithmic transformation as needed to ensure normality of skewed variables. Student’s t-test was performed to assess the differences between two independent means. Differences in the variables within the same group, before and after BS, were analysed by repeated-measures ANOVA. The comparison of quantitative variables of depressive symptoms and QoL between the groups was based on analysis of variance adjusted for the patients’ sex, age and BMI (ANCOVA). In addition, the effect size of the mean comparisons was estimated with the standardized Cohen’s d coefficient (|d| > 0.20 was considered low, |d| > 0.5 was considered moderate and |d| > 0.8 was considered high) [42]. The level of significance was set at *p* < 0.05 for the main effects and the interaction. Relationships between clinical and metabolic data, different serum proteins or miRNA expression with the psychometric test results were analysed via Spearman’s correlation test. All statistical analyses were performed using SPSS statistical software 26.0 (SPSS Inc., Chicago, IL, USA).

## 3. Results

### 3.1. Characteristics of Participants

Differences in clinical and metabolic variables before and 8 years after BS between the RYGB/BPD and SG groups are summarized in Table 1. Patients with poorer metabolic control and gastroesophageal reflux disease underwent RYGB/BPD. BS improved most of the anthropometric and biochemical variables in both experimental groups (Table 1). 

Table 2 summarizes clinical and biochemical characteristics of patients classified by their response 8 years after BS (GR and NR). At baseline, significant differences were only found in terms of insulin and HOMA-IR, with the GR group showing higher values than the NR group. The GR group showed better anthropometric and metabolic values after BS than the NR group. 

### 3.2. Quality of Life and Depression at 8-Year Follow-Up after BS

No statistically significant differences were found between the two groups of BS techniques (RYGB/BPD and SG) in neither the level of depression (BDI-II), nor any of the perceived QoL scales (SF-36). Table 3 includes the ANCOVA (adjusted for sex, age and BMI) comparing SF-36 and BDI-II scores between the two groups based on their outcome (GR vs. NR). The GR group showed higher scores on the SF-36 scales of physical functioning (*p* = 0.039), role functioning—physical (*p* = 0.008), role functioning—emotional (*p* = 0.013) and body pain (*p* = 0.029), as well as higher scores on the global general health (*p* = 0.020), than the NR group. Analyses of the depression state did not show statistically significant differences between the NR and GR groups, although a trend was observed (Supplementary Figure S1).

### 3.3. Association between Serum Protein Levels before and after Surgery vs. Psychological Variables

**Figure 1:**
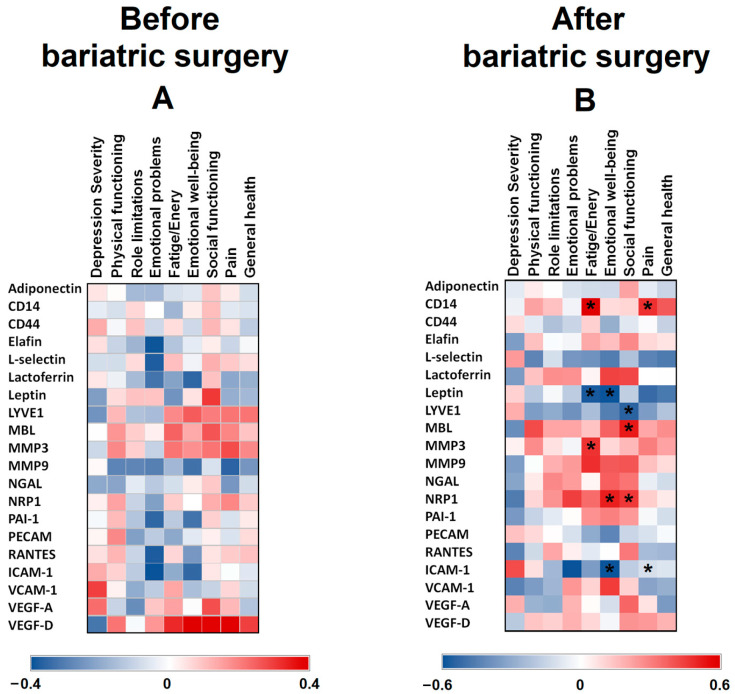
Heat maps representing associations between SF-36 and BDI-II scores vs. serum protein levels in patients with class III obesity before (**A**) and 8 years after bariatric surgery (**B**). The Spearman correlation is displayed on a colour scale from blue (negative correlation) to red (positive correlation). * Significant correlations (*p* < 0.05).

Since the relation between different adipocytokines and psychological variables have not been well studied in relation to the mental well-being of patients with class III obesity, these proteins were analysed at baseline and 8 years after BS (Figure 1). No significant correlations were found between psychometric tests results and the levels of different basal proteins (before BS) (Figure 1A). Figure 1B shows the heatmap of the correlations between protein levels 8 years after BS and the mental health of the patients. The Fatigue/Energy measurement positively correlated with CD14 and MMP3 (*p* = 0.012 and *p* = 0.048, respectively), whereas it negatively correlated with leptin (*p* = 0.025). Emotional well-being negatively correlated with leptin (*p* = 0.023) and ICAM-1 (*p* = 0.048), and positively with NRP1 (*p* = 0.038). Social functioning positively correlated with MBL (*p* = 0.023) and NRP1 (*p* = 0.046), and negatively with LYVE1 (*p* = 0.038). Pain measurement positively correlated with CD14 levels (*p* = 0.039), and negatively with ICAM-1 levels (*p* = 0.031).

### 3.4. Association between Serum Protein Levels before and after Surgery vs. Bariatric Surgery Outcome

**Figure 2:**
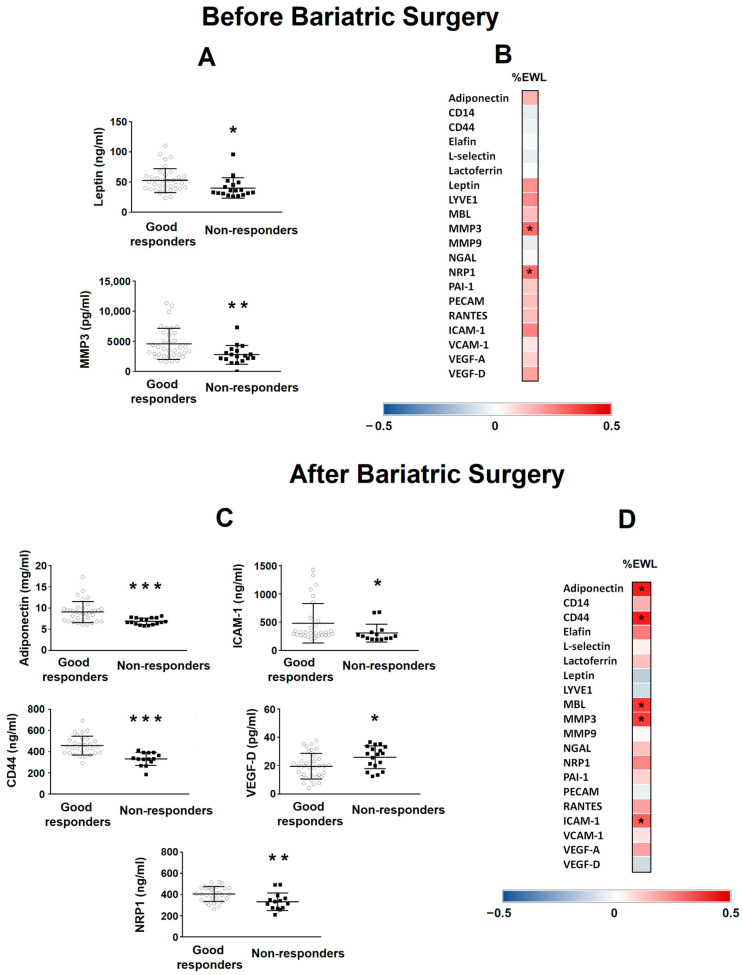
Levels of different proteins in patients with class III obesity before and 8 years after bariatric surgery. (**A**) Levels of basal serum leptin and MMP3 in patients with class III obesity classified depending on their outcome after bariatric surgery (good responders vs. non-responders). Data are expressed as the mean  ±  SD. Statistical significance is expressed as: * *p* < 0.05; ** *p* < 0.01. (**B**) The heat map represents the associations between basal serum protein levels and the percentage of excess weight lost (%EWL) 8 years after bariatric surgery. * Significant correlations (*p* < 0.05). (**C**) Levels of serum adiponectin, ICAM-1, CD44, VEGF-D and NRP1 in patients with class III obesity 8 years after bariatric surgery classified depending on their outcome after bariatric surgery (good responders vs. non-responders). Data are expressed as the mean  ±  SD. Statistical significance is expressed as: * *p* < 0.05; ** *p* < 0.01; *** *p* < 0.001. (**D**) The heat map represents the associations between serum proteins levels 8 years after bariatric surgery vs. %EWL 8 years after bariatric surgery. * Significant correlations (*p* < 0.05). The Spearman correlation is displayed on a colour scale from blue (negative correlation) to red (positive correlation).

When these basal proteins were analysed according to BS outcome (Figure 2A), only leptin and MMP3 showed differences between the GR and NR groups, with the lowest levels in the NR group (*p* = 0.028 and *p* = 0.009, respectively). Figure 2B shows the heatmap of correlations between protein levels before BS and the %EWL of patients. Only MMP3 (*p* = 0.292) and NRP1 (*p* = 0.291) positively correlated with the %EWL of patients. Figure 2C shows the differences between the GR and NR groups in different protein levels 8 years after BS. Adiponectin (*p* = 0.001), ICAM-1 (*p* = 0.017), CD44 (*p* = 0.001) and NRP1 (*p* = 0.041) levels were lower in the NR group compared with the GR group, whereas the VEGF-D level was higher (*p* = 0.045). Figure 2D shows the heatmap of the correlations between protein levels 8 years after BS and the %EWL of patients. Adiponectin (*p* = 0.436), CD44 (*p* = 0.469), MBL (*p* = 0.392), MMP3 (*p* = 0.379) and ICAM-1 (*p* = 0.310) positively correlated with the %EWL of patients.

### 3.5. miRNA Levels as a Biomarker Predictor of Intervention Success in Patients with Class III Obesity

**Figure 3:**
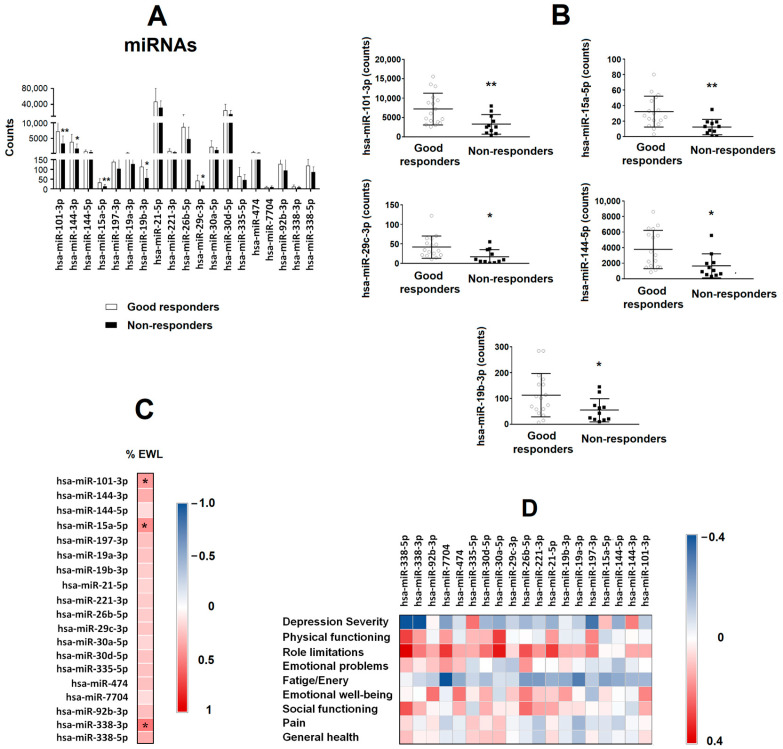
Serum miRNA levels as a predictive biomarker of bariatric surgery success in patients with class III obesity. (**A**) Different miRNAs related to depression and anxiety were evaluated by sequencing in the serum of patients with class III obesity before they underwent bariatric surgery. Patients were classified depending on their outcome after bariatric surgery (good responders vs. non-responders). Data are expressed as the mean  ±  SD. * *p* < 0.05; ** *p* < 0.01. (**B**) Levels of basal serum hsa-miR-101-3p, hsa-miR-15a-5p, hsa-miR-19b-3p, hsa-miR-29c-3p and hsa-miR-144-5p in patients with class III obesity, classified depending on their outcome after bariatric surgery (good responders vs. non-responders). Data are expressed as the mean  ±  SD. * *p* < 0.05; ** *p* < 0.01. (**C**) The heat map represents the associations between basal serum miRNAs levels and the percentage of excess weight lost (%EWL) 8 years after bariatric surgery. * Significant correlations (*p* < 0.05). (**D**) The heat map represents the association between basal serum miRNA levels and SF-36 and BDI-II scores. The Spearman correlation is displayed on a colour scale from blue (negative correlation) to red (positive correlation).

Figure 3A shows different depression/anxiety-related miRNAs at basal levels obtained from serum samples from both groups (GR and NR).

Lower levels of hsa-miR-101-3p (*p* = 0.008), hsa-miR-15a-5p (*p* = 0.005), hsa-miR-29c-3p (*p* = 0.016), hsa-miR-144-3p (*p* = 0.018) and hsa-miR-19b-3p (*p* = 0.046) were found in the NR group compared with the GR group (Figure 3B). No significant changes were found on the rest of miRNAs analysed. The heatmap (Figure 3C) shows the correlations between miRNAs at basal level and the %EWL of the patients. The %EWL was positively correlated with hsa-miR-101-3p (*p* = 0.040), hsa-miR-15a-5p (*p* = 0.022) and hsa-miR-338-3p (*p* = 0.003). The variable that was associated with the response to BS (good responders = 0 and non-responders = 1) in a logistic regression model was hsa-miR-19b-3p (β = 0.019, *p* = 0.041, OR = 1.019, 95% IC = 1.001–1.037) after adjusting for sex, BMI and the other four miRNAs (hsa-miR-101-3p, hsa-miR-15a-5p, hsa-miR-29c-3p, hsa-miR-144-3p and hsa-miR-19b-3p).

To analyse the association between those basal miRNAs and the mental health of patients 8 years after BS, correlations were performed between miRNAs and the results of the different psychometric tests (Figure 3D). No significant associations were found.

## 4. Discussion

In this study, we sought to determine whether the %EWL is associated with QoL or depressive symptomatology in patients with class III obesity at the 8-year follow-up after BS, as well as its possible association with different serum proteins and miRNAs. The GR group showed higher scores on the SF-36 scales of physical functioning, role functioning—physical, role functioning—emotional and body pain, as well as higher scores on global general health, than the NR group. On the other hand, different adipocytokines showed significant correlations with patients’ mental health and the %EWL 8 years after BS. In addition, the levels of several proteins were lower in the NR group at baseline and 8 years after BS. Moreover, different miRNAs were lower in the NR group, with hsa-miR-19b-3p being the miRNA most closely associated with the response to BS.

There are many studies focusing on the mental health of patients with obesity and how it changes after BS. The interesting contribution of this study is to analyse the data according to the percentage of weight loss instead of an analysis by surgical approach. Patients with a favourable outcome at the 8-year follow-up showed greater improvement in depression severity and in most of the QoL domains (such as physical functioning, role—physical, body pain, role—emotional, global general health and global index of QoL) than those with a poor outcome. These findings reinforce the fact that a successful BS can yield not only weight loss and stabilization of metabolic parameters, but also mental health and social improvements [16,17,18,43]. These results suggest that success in BS is associated with the recovery of daily activities that were previously physically and mentally limited, recovery and participation in social activities, less interference of pain with activities and household responsibilities, improvement of mood state and depressive symptoms and, in general, with a better perception of patients’ own level of health and QoL. Likewise, in the group with poor outcome, the frustration of not achieving BS success and meeting weight loss expectations could also accentuate their psychological distress and poor perception of QoL. However, it seems that all findings follow the same path, suggesting that the improvement in QoL and depressive symptoms depends on the outcome of the BS.

In obesity, the presence of chronic low-grade systemic inflammation is well known. Some pro-inflammatory cytokines are associated with depression [25]. However, there are other serum proteins that are less studied and secreted by adipose tissue, which may also be associated with the regulation of inflammation, directly or indirectly, and other processes involved in mechanisms underlying depression. In the present study, different serum proteins were lower in the NR group compared with the GR group, mainly after BS. Moreover, most of those proteins positively correlated with the mental health of patients after BS. One interesting fact is the increased level of VEGF-D 8 years after the intervention in the NR group, who were the ones with the worst results in the psychometric tests. This angiogenic factor is elevated in the serum of overweight and obese individuals [44]. In obesity development, it has been shown that VEGF-D could be released in order to ameliorate the adipose tissue inflammation [44]. The higher level of VEGF-D found in the NR group could be due to an inadequate weight loss, per se. Regarding NRP1, the NR group showed lower levels than the GR group, and the correlation with the emotional well-being and social functioning was positive, which can explain why the NR group performed worse in the psychometric tests. This protein seems to protect against obesity and metabolic syndrome [45]. Another interesting fact was the decrease in adiponectin levels found in the NR compared with the GR group, since these have been related to atypical depression [46]. On the other hand, ICAM-1 is a pro-inflammatory molecule, which might be a useful biomarker for depression associated with vascular inflammation. This protein was negatively associated with emotional well-being. This is consistent with the hypothesis that depression can be secondary to vascular inflammation [47]. Together, our results show a group of proteins with different functions, some of them not clearly associated with the regulation of obesity, which seem to be associated with a favourable outcome at the 8-year follow-up. However, further in-depth investigations into their role in the regulation of weight and mental well-being of these patients are needed.

We were also able to identify a group of proteins, some of the above and others such as CD14, leptin, MBL and MMP3, which could be used as an indirect biomarker of mental illnesses, and which link different aspects of inflammation with the results of psychometric tests. CD14 is a surface antigen, which participates in the innate immune response to bacterial lipopolysaccharide. In this context, serum MBL is an important element in the innate immune system and recognizes the mannose and *N*-acetylglucosamine of a wide range of microbial microorganisms. These proteins would increase the systemic inflammation in response to different microbial components and may lead to neuroinflammation in the central nervous system [48]. This neuroinflammation is also induced by MMP3, an endogenous neuronal activator of microglia [49]. Also, plasma leptin has been negatively correlated, as in our case, with symptoms of anxiety and depression [50].

Regarding miRNAs, they were analysed to understand their predictive ability in patients with obesity undergoing different surgical approaches. Some studies show similarities with our data. For instance, hsa-miRNA-144-5p was downregulated in the NR group, who showed a higher percentage of depression. This has been also found in depressive patients compared with healthy individuals [51]. Looking at the miRNA serum expression per group of patients, most of them presented lower values in the NR vs. GR group, which may also explain the worse performance of this group during psychometric tests and their non-response to the intervention 8 years after. A previous study showed that lower levels of mRNAs were found in patients with depression [41]. The relationship between depression and obesity is reciprocal [18]. In this context, the significant miRNAs found in our study that are related to depression/anxiety could also be associated with the regulation of obesity. For example, several miRNAs, such as miR-144-5p, miR-15a-5p and miR-29c-3p, are associated with a response to a low-fat diet and with several metabolic processes, including glucose and lipid metabolism, adipocyte development and adipose tissue physiology, inflammatory pathways and weight gain [52]. Moreover, hsa-miR-15a-5p binds to CCND1 [53], which is a molecule regulated by FTO [54], a gene identified as a risk gene for obesity. Regarding hsa-miR-19b-3p, its expression changed after bariatric surgery, suggesting an association with the weight loss [55]. In our study, we have found that has-mir-19b-3p was the miRNA more closely associated with a good response 8 years after BS. Serum miR-19b-3p upregulation was found to inhibit the lipopolysaccharide-induced expression of pro-inflammatory cytokines IL-6 and TNF-α [56]. Another study found that miR-19b-3p attenuated the inflammatory injury [57], and that the upregulation of circulating miR-19b-3p suppressed fibrogenesis in the liver by regulating inflammation [58]. The interesting contribution of our study is the finding that most of these miRNAs could be used as predictors of the response to BS in a non-invasive way, which is the main goal of the personalized medicine approach, and more specifically, we could suggest that miR-19b-3p, an miRNA related to depression/anxiety, would be involved in achieving a good response to BS.

The main limitation of this retrospective study is the lack of psychometric tests before BS, which would be interesting to have had in order to know if depressive symptoms observed in the non-responders group were already present before BS and if therefore, these patients did not achieve a positive response after BS. Moreover, the number of participants is quite limited, so results should be further validated in additional cohorts of patients. Overall, although some caution should be exercised with the results obtained, they can give us preliminary information of some relevance. Another limitation of the study is the non-use of the %TWL instead of the %EWL to classify patients. In this study, we wanted to show whether the loss of excess weight needed to be considered normal weight can be related to QoL/depression, miRNAs related to the latter pathology or adipocytokines. The perception that obesity is still present or that one’s weight is in the normal range (BMI greater than or equal to 18.5 to 24.9 kg/m^2^), and not only the amount of weight loss, is of great importance for the state of depression [14]. In this regard, it is true that recent guidelines recommend the use of the %TWL [59] because this index seems to be less influenced by the baseline BMI during short-term follow-ups after SG and RYGB [60]. However, to date, there are no perfect indices for reporting weight loss outcomes after BS that are completely independent of the baseline BMI. When the %EWL is used, patients with a lower BMI have significantly “better” weight loss outcomes [60]. However, from 9 months after surgery, results using the %TWL have shown a significant trend of better outcomes for patients with higher BMI, especially in the case of RYGB [60]. Furthermore, the cut-off point for the %TWL is unclear, with some studies using 20% [61,62,63,64] and others using 25% [65,66,67]. A comparison of the obtained results relating to QoL/depression, miRNAs and adipocytokines with these two indices, %EWL vs. %TWL, is not the aim of the present study. This needs to be further analysed in other studies and with a larger number of participants. On the other hand, recent studies show that the information provided by body composition techniques for determining body fat percentage, such as bioelectrical impedance analysis, dual-energy X-ray absorptiometry, plethysmography or magnetic resonance imaging [68], may be more relevant than that provided by BMI. These new techniques make it possible to detect the presence of a condition with important functional implications, consisting of the simultaneous presence of excess adiposity and a deficit in skeletal muscle mass and function (sarcopenia), which is defined as sarcopenic obesity [69,70]. This medical condition is highly correlated with an increased risk of obesity-related comorbidities [69] and is associated with increased mortality [71]. However, there are hardly any studies using these techniques to analyse body composition and QoL in long-term follow-up of patients undergoing BS [72].

## 5. Conclusions

Taking into consideration all this information, we can outline two main conclusions. First, the mental health of the individual after BS is limited by the success of the intervention. Second, we showed that the expression of basal serum miRNAs related to depression/anxiety could predict the success of bariatric surgery in patients with class III obesity. Further investigation would be appropriate to assess whether these proteins and miRNAs before bariatric surgery could indicate whether class III obesity could lead to symptoms of depression/anxiety.

## Figures and Tables

**Table 1:** Clinical and metabolic variables for each bariatric surgery type, before (white rows) and 8 years after (grey rows) the intervention.

	RYGB/BPD	SG	*p*-Value
*n* (male/female)	41 (6/35)	49 (9/40)	0.64
Age at the time of surgery (years)	42.4 ± 9.8	44.3 ± 9	0.34
%EWL	58.5 ± 36.6	52.8 ± 25.7	0.071
%TWL	30.1 ± 13.4	24.7 ± 12.1	0.003
BMI (kg/m^2^)	50.1 ± 7.8	48.6 ± 7.6	0.03
	34.8 ± 7.3 ***	36.1 ± 6.1 ***	0.66
Waist (cm)	133.7 ± 15.5	130.5 ± 15.1	0.05
	103.3 ± 18.1 ***	104.2 ± 15.5 ***	0.74
Hip (cm)	145.8 ± 17.2	144.5 ± 12.3	0.17
	117.4 ± 17 ***	121.5 ± 12.5 ***	0.27
Waist/hip ratio	0.93 ± 0.15	0.9 ± 0.09	0.53
	0.87 ± 0.09 *	0.86 ± 0.09 *	0.26
SBP (mmHg)	138.5 ± 19.8	133 ± 17.6	0.23
	118 ± 16.1 ***	130.9 ± 23.6	0.002
DBP (mmHg)	82.9 ± 12.4	80.8 ± 9	0.42
	67.7 ± 9.3 ***	72 ± 12.6 ***	0.02
Glucose (mg/dl)	111.9 ± 44.1	97.9 ± 27	0.01
	81.4 ± 9.9 ***	84.1 ± 9 ***	0.45
Insulin (mg/dl)	20.8 ± 11.7	15.9 ± 12.1	0.03
	7.8 ± 6.1 ***	9.9 ± 5.3 ***	0.10
HOMA-IR	6.2 ± 5	4.1 ± 3.4	0.003
	1.65 ± 1.6 ***	2.12 ± 1.3 ***	0.15
Triglycerides (mg/dl)	136.9 ± 89	118.6 ± 53.8	0.17
	87.6 ± 35.4 ***	89.8 ± 40.5 ***	0.62
Cholesterol (mg/dl)	190.2 ± 41.8	178 ± 34.7	0.16
	164.1 ± 40.2	197.6 ± 39.4	<0.001
HDL-c (mg/dl)	46.3 ± 14.4	44.3 ± 13.7	0.47
	61.9 ± 16.9 ***	66.8 ± 16.5 ***	0.18
LDL-c (mg/dl)	113.5 ± 31.2	110.6 ± 33.6	0.92
	84.5 ± 31.1 ***	112.3 ± 32.6	<0.001

Data are means ± SD. %EWL, percentage of excess weight lost; %TWL, percentage of total weight loss; BMI, body mass index; SBP, systolic blood pressure; DBP, diastolic blood pressure; HDL-c, high-density lipoprotein cholesterol; LDL-c, low-density lipoprotein cholesterol. *p*-value column represents the *p*-value between types of bariatric surgery. The significant effect of time (basal vs. 8 years after bariatric surgery) is represented as * *p* < 0.05 and *** *p* < 0.005.

**Table 2:** Clinical and metabolic variables split by good responders vs. non-responders to bariatric surgery, before (white rows) and 8 years after (grey rows) bariatric surgery.

	Good Responders	Non-Responders	*p*-Value
*n* (male/female)	60 (9/51)	30 (6/24)	0.55
Type of surgery (RYGB-BPD/SG)	60 (28/32)	30 (12/18)	0.35
Age at the time of surgery (years)	42.5 ± 10.2	45.3 ± 7.5	0.19
%EWL	71.0 ± 14.7	23.0 ± 34.8	<0.001
%TWL	33.8 ± 7.3	13.8 ± 12.6	<0.001
BMI (kg/m^2^)	48.8 ± 6.7	50 ± 9.3	0.55
	32.2 ± 4.6 ***	42.2 ± 4.9 ***	<0.001
Waist (cm)	132.2 ± 15.7	131.6 ± 14.6	0.87
	98.1 ± 15.9 ***	116.3 ± 12.2 ***	<0.001
Hip (cm)	145.9 ± 13	143.57 ± 17.6	0.49
	112.8 ± 12.1 ***	132.7931 ± 11.3 **	<0.001
Waist/hip ratio	0.91 ± 0.1	0.94 ± 0.2	0.34
	0.85 ± 0.09 ***	0.9 ± 0.1	0.48
SBP (mmHg)	135.4 ± 19.2	134.6 ± 18.1	0.87
	116.6 ± 15.7 ***	140 ± 23.7	<0.001
DBP (mmHg)	81.8 ± 10.7	79.6 ± 9.1	0.38
	67.1 ± 10.4 ***	75.4 ± 11.2	0.005
Glucose (mg/dl)	108.1 ± 41.7	97 ± 19.9	0.11
	81 ± 6.9 ***	87.2 ± 12.4 **	0.02
Insulin (mg/dl)	20.5 ±13.8	14.5 ± 7.3	0.05
	7.6 ± 5.8 ***	11.5 ± 4.9 *	0.003
HOMA-IR	5.9 ± 5	3.6 ± 2.2	0.01
	1.6 ± 1.5 ***	2.5 ± 1.3 *	0.004
Triglycerides (mg/dl)	134.2 ± 78.7	112.9 ±55.4	0.20
	79.2 ± 29.7 ***	108.5 ± 46 **	0.004
Cholesterol (mg/dl)	184.9 ± 40.9	180.5 ± 33.7	0.61
	181.2 ± 44.5	185.6 ± 41.3	0.72
HDL-c (mg/dl)	44.8 ± 13.6	45.3 ± 14.7	0.95
	66.6 ± 17.9 ***	59.9 ± 13.4 ***	0.05
LDL-c (mg/dl)	110.9 ± 34.4	113.7 ± 29.2	0.68
	99.2 ±35.1 *	103.6 ±33.5 *	0.52

Data are means ± SD. %EWL, percentage of excess weight lost; %TWL, percentage of total weight loss; BMI, body mass index; SBP, systolic blood pressure; DBP, diastolic blood pressure; HDL-c, high-density lipoprotein cholesterol; LDL-c, low-density lipoprotein cholesterol. *p*-value column represents the *p*-value between types of bariatric surgery. The significant effect of time (basal vs. 8 years after bariatric surgery) is represented as * *p* < 0.05, ** *p* < 0.01 and *** *p* < 0.005.

**Table 3:** Comparison of depressive symptoms and quality of life based on the bariatric surgery outcome: ANCOVA adjusted by sex, age and BMI.

	Good Responders (*n* = 30)		Non-Responders (*n* = 23)			
	*Mean*	*SD*	*Mean*	*SD*	*p*	*|d|*
SF-36 Physical functioning	72.32	26.95	54.07	27.95	**0.039** *	**0.66** ^†^
SF-36 Role—physical	74.71	40.97	37.52	42.80	**0.008** *	**0.89** ^†^
SF-36 Body pain	59.63	20.80	43.27	24.48	**0.029** *	**0.72** ^†^
SF-36 General health	70.97	23.29	59.75	22.15	0.129	**0.51** ^†^
SF-36 Vitality	49.93	25.19	40.06	22.85	0.187	0.41
SF-36 Social functioning	82.04	23.21	65.12	28.51	0.054	**0.65** ^†^
SF-36 Role—emotional	84.02	39.65	51.28	43.25	**0.013** *	**0.79** ^†^
SF-36 Mental health	59.79	24.39	53.57	26.10	0.449	0.25
SF-36 Global general health	69.24	22.63	51.39	24.68	**0.020** *	**0.75** ^†^
BDI-II Total	12.44	13.52	22.44	15.91	**0.040** *	**0.68** ^†^

Note. SD: standard deviation. * Bold: significant comparison. ^†^ Bold: effect size in the mild to large range.

## Data Availability

The data presented in this study are available on request from the corresponding author. The data are not publicly available due to ethical reasons.

## Supplementary Materials

The following supporting information can be downloaded at: https://www.mdpi.com/article/10.3390/nu15194109/s1, Figure S1: Pie charts representing the severity of depression at 8-year follow-up after bariatric surgery in percentage out of the total number of patients (good responders vs. non-responders groups).
