# Supplementary material for: The Relationship between Depressive Symptoms, Quality of Life and miRNAs 8 Years after Bariatric Surgery
Source: Nutrients. 2023 Sep 22;15(19):4109. doi: 10.3390/nu15194109 (PMC10574314; doi:10.3390/nu15194109)
Supplement: Supplementary file 1 [file nutrients-15-04109-s001.zip › nutrients-2604252-supplementary.pdf]

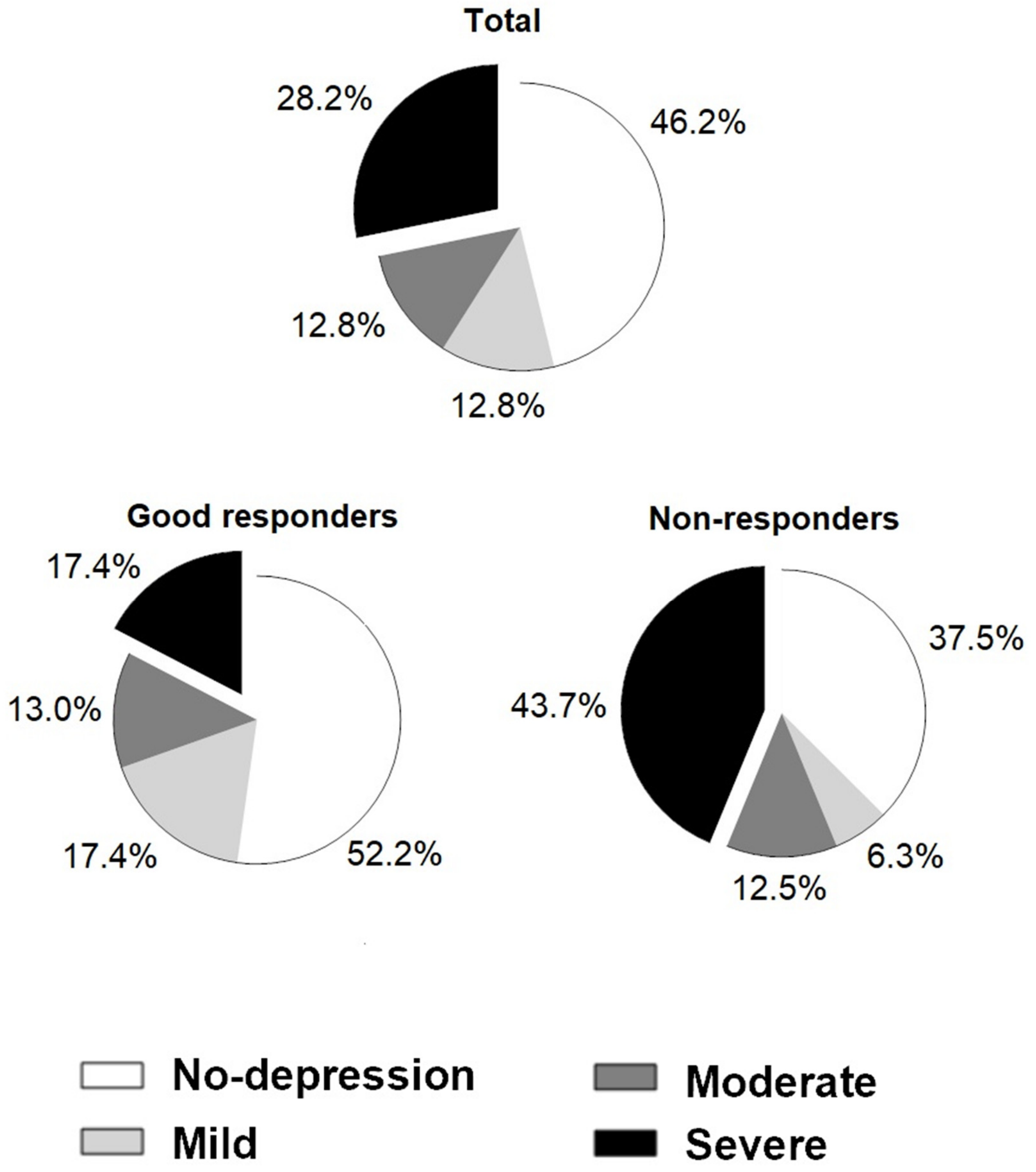

Figure S1. Pie charts representing the severity of depression at 8-year follow-up after bariatric surgery in percentage out of the total number of patients (good responders vs. non-responders groups).
